# Human Metabolites of Cannabidiol: A Review on Their Formation, Biological Activity, and Relevance in Therapy

**DOI:** 10.1089/can.2015.0012

**Published:** 2016-03-01

**Authors:** István Ujváry, Lumír Hanuš

**Affiliations:** ^1^ *i*Kem BT, Budapest, Hungary.; ^2^ Institute for Drug Research, Hebrew University Medical Faculty, Jerusalem, Israel.

**Keywords:** biological activity, cannabidiol, metabolites, pharmacokinetics, synthesis

## Abstract

Cannabidiol (CBD), the main nonpsychoactive constituent of *Cannabis sativa*, has shown a wide range of therapeutically promising pharmacological effects either as a sole drug or in combination with other drugs in adjunctive therapy. However, the targets involved in the therapeutic effects of CBD appear to be elusive. Furthermore, scarce information is available on the biological activity of its human metabolites which, when formed in pharmacologically relevant concentration, might contribute to or even account for the observed therapeutic effects. The present overview summarizes our current knowledge on the pharmacokinetics and metabolic fate of CBD in humans, reviews studies on the biological activity of CBD metabolites either *in vitro* or *in vivo*, and discusses relevant drug–drug interactions. To facilitate further research in the area, the reported syntheses of CBD metabolites are also catalogued.

## Introduction

**FIG. 1.**
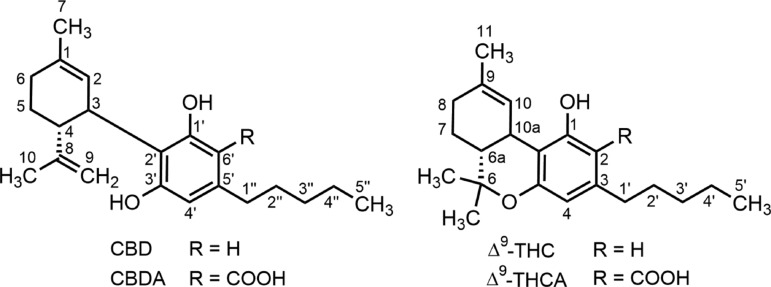
Chemical structures and numbering system for CBD and Δ^9^- THC type cannabinoids. CBD, cannabidiol; THC, tetrahydrocannabinol.

Cannabidiol (CBD; Fig. 1) is one of the chemically and phytogenetically related phenolic terpenes derived from hemp (*Cannabis sativa* L). It was first obtained in pure form in 1940 simultaneously from fiber-type American hemp^1^ and from psychotropic Egyptian hashish.^2^ The chemical structure of CBD was determined by Mechoulam and Shvo in 1963.^3^ CBD is one of the 142 phytocannabinoids that have been isolated so far from hemp.^4^ Strictly speaking, however, CBD is an artifact: the genuine natural product is cannabidiolic acid (CBDA; Fig. 1), which under the influence of heat is decarboxylated into CBD in the plant material. Likewise, another major phenolic terpene of hemp, Δ^9^-tetrahydrocannabinol (THC)^5^ is formed from the corresponding carboxylic acid (THCA; Fig. 1).

Although CBD was isolated and characterized first, THC has been investigated more thoroughly: THC is responsible for the unique psychoactivity of marijuana, or cannabis, which is an internationally controlled substance, nevertheless widely used for recreational purposes or, more recently, for self-medication.^6^ Synthetic THC has been available for three decades as a medicine, and pharmaceutical-grade herbal cannabis, as well as formulations of cannabis extracts containing THC and CBD in well-defined ratios, has also been registered as medicines in several countries (see chapters of Part 3 of Pertwee^6^). Due to its unique psychoactivity and therapeutic potential, both associated with the activation of cannabinoid (CB) receptors, as well as for forensic reasons, the pharmacokinetics and pharmacodynamics of THC is much better understood than those of the nonpsychoactive CBD, which for decades has been a neglected phytocannabinoid.

The chemistry and pharmacology of CBD, as well as the various molecular targets, including CB receptors and other components of the endocannabinoid system it interacts with, have adequately been reviewed,^7–11^ while the pharmacology of CBD analogs, with emphasis on anti-inflammatory effects, was the subject of a recent overview.^12^

In the recent decade, preclinical studies, human case reports, and a plethora of anecdotal accounts, recognizing the relative safety of CBD, have prompted the exploration of the therapeutic potential of CBD against a range of diseases.^13–18^ In particular, the promise of CBD in treating cancer and drug-resistant epilepsy in children has recently brought this natural product into the focus of the scientific community, clinicians, the media, as well as politicians and regulatory agencies.^19–24^ Consequently, the US Food and Drug Administration and the European Medicines Agency have granted CBD preparations the “Orphan Drug” designation for use in the treatment of epilepsy in children (Dravet and Lennox-Gastaut syndromes) and neonatal asphyxia, and clinical trials sponsored by GW Pharma Ltd. have been started in these indication areas.^25,25a,25b^

While some information on the pharmacokinetics of CBD in experimental animals and humans is available,^26–29^ the biological activity of CBD metabolites has received scant attention.^30^ The purpose of this review is to summarize our current knowledge of the human pharmacokinetics of CBD with particular emphasis on the biological properties of established or putative human metabolites of CBD. We also indicate several gaps in our knowledge on CBD metabolites, which should be filled by further research that aims to expand the therapeutic use of CBD-based medications. Forensic studies reporting on CB levels as detected in the urine, blood, or saliva of smokers of cannabis cigarettes or of users of various medicinal cannabis preparations have been excluded (for recent reviews, see Huestis^28^ and Huestis and Smith^29^). To facilitate further research in the area, the synthetic routes reported for CBD metabolites and their close structural analogs are also catalogued.

## Human Pharmacokinetics of CBD Upon Various Administration Routes

**FIG. 2.**
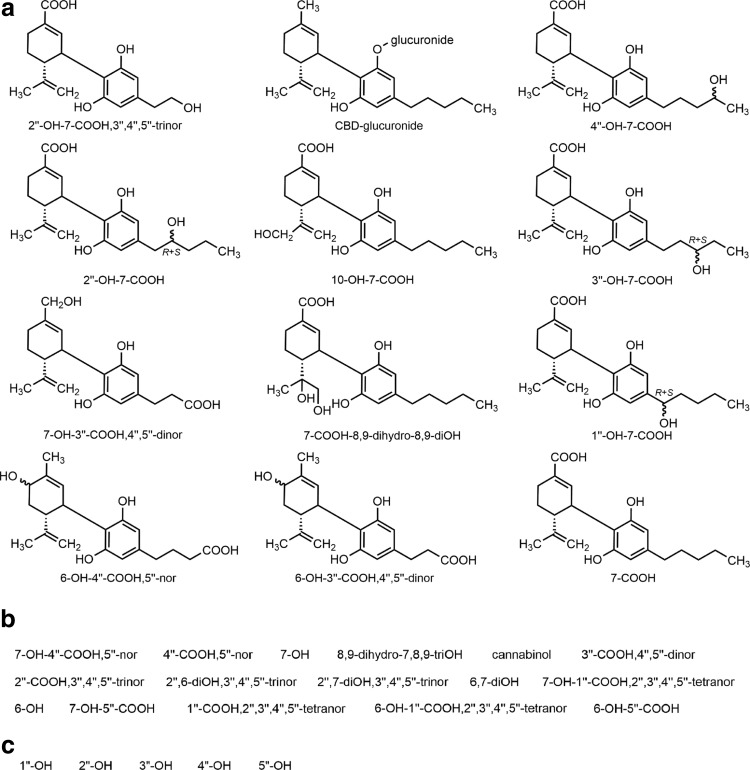
**(a)** Chemical structures of major (>1%) metabolites of CBD identified by GC-MS in anhydrolyzed urine of a dystonic patient treated chronically with the drug.^59^ The compounds are listed according to their relative amounts (decreasing from upper left to lower right) based on peak areas of the gas chromatogram. Additional major metabolite was Δ^8^-THC (1.97%; structure not shown). In the structural diagrams, wavy bonds indicate stereogenic center with *R*+*S* indicating that the two epimers were chromatographically separated; otherwise, stereochemistry is unknown. **(b)** List of minor (<1%) urinary metabolites present in the urine of the same patient; additional excreted compound present was Δ^9^-THC (0.69%), which has also been identified in artificial gastric juice.^128^ For numbering, see Figure 1. The chemical structures of the minor metabolites are given in Supplementary Figure S1. **(c)** CBD metabolites identified in human liver microsomal preparation.^60^ Their chemical structures are given in Supplementary Figure S1. For convenience, the shorthand notation adopted here indicates only the newly formed functional group(s) present in the metabolite of CBD.

Extensive studies in animals, including rodents and the dog, indicate that a large portion of the administered CBD is excreted intact or as its glucuronide.^26,27^ Due to extensive Phase I metabolism, the pharmacokinetics of CBD is complex and the bioavailability of oral CBD is low across species.^26–29^ In general, the most abundant metabolites are hydroxylated 7-COOH derivatives of CBD (Fig. 2) that are excreted either intact or as glucuronide conjugates. The route of administration affects the pharmacokinetics of CBD and high intra- and intersubject variability is common in humans as the following paragraphs demonstrate.

In five individuals each smoking a single cigarette containing ∼19 mg [^2^H]CBD, the average peak blood plasma level of CBD was 110 ng/mL (range: 42–191 ng/mL) recorded at 3 min postdose; the mean half-life was 31±4 h and the average systemic availability was 31% (range: 11–45%).^31^

In an early study with healthy volunteers who were given 20 mg [^3^H]CBD by intravenous (i.v.) injection, 7-COOH-CBD was the most abundant metabolite in the plasma, while 7-OH-CBD was only a minor biotransformation product (in the original publication, the compounds are referred to as 11-carboxy-CBD and 11-hydroxy-CBD, respectively).^32^ In the urine, unchanged CBD and, to a lesser extent, conjugated CBD were the main excretion products and about 16% of the total radioactivity was eliminated in 72 h by this route of excretion. It was also observed that 33% of the total radioactivity, again mostly unchanged CBD accompanied by several oxygenated metabolites, including mono- and dihydroxylated and monocarboxylic derivatives of CBD, was excreted in the feces within 72 h. In a subsequent and more detailed investigation, five young marijuana smokers were given 20 mg [^2^H]CBD by i.v. injection.^31^ At 3 min following drug administration, the CBD plasma levels peaked at 686 ng/mL (range: 356–962 ng/mL), which rapidly dropped to 48 ng/mL (range: 37–61 ng/mL) after 1 h; the mean half-life was 24±6 h.

In 12 subjects, oral administration of chocolate cookies spiked with a blend of 40 mg CBD+20 mg THC resulted in low peak plasma levels of ∼5 ng/mL for each drug at 1.5–3 h.^33^ Similar low peak plasma levels with a mean of 0.93 ng/mL (range: 0.3–2.6 ng/mL) were noted in 24 volunteers 1 h after oral ingestion of gelatin capsules with cannabis extract containing 5.4 mg CBD+10 mg THC.^34^ Interesting results were obtained from experiments in which capsules filled with either unheated or heated cannabis extracts containing 10 mg THC_total_ (THC+THCA) and 10–15 mg CBD_total_ (CBD+CBDA) estimated when fresh: pharmacokinetic analysis of the blood of patients ingesting two such capsules showed mean peak plasma CBD concentrations four times higher in the unheated extract than in the heat-treated extract (1.24 ng/mL at 1.17 h vs. 0.30 ng/mL at 0.83 h, respectively).^35^ The results suggest that the use of unheated cannabis extract rich in acidic phytocannabinoids may beneficially affect the uptake and metabolism of CBD or other phytocannabinoids.

In another investigation, repeated oral administration of daily doses of 700 mg of CBD to 14 Huntington's disease patients did not result in elevated mean blood concentrations; in a 6-week trial, plasma levels of the drug remained in a relatively constant but low range of 5.9–11.2 ng/mL throughout the trial, averaged 1.5 ng/mL 1 week after CBD administration was discontinued and virtually undetectable by gas chromatography coupled with mass spectrometry (GC-MS) thereafter; the elimination half-life of CBD ranged from 2 to 5 days.^36,37^ In this study, CBD was found to be neither symptomatically beneficial nor toxic. During a functional magnetic resonance imaging investigation of the effects of THC and CBD on regional brain function in 15 healthy volunteers, respective mean blood concentrations of 4.7±7 and 17±29 ng/mL of CBD were recorded at 1 and 2 h following a single oral dose of 600 mg CBD.^38^

A recent study examined the safety and pharmacokinetics of 400 and 800 mg of CBD coadministered with various doses of the potent opioid analgesic fentanyl to 17 healthy individuals.^39^ In a representative session, 3 h after the oral administration of 800 mg of CBD and 2 h after fentanyl injection (0.5 μg/kg i.v.), the highest plasma concentration of CBD was 221±36 ng/mL; the mean peak urinary CBD concentration was recorded at 4 h after CBD intake and estimated to be 3.7 ng/mL.

As a part of a series of trials with “Cannabis Based Medicine Extracts” such as Sativex^®^, the pharmacokinetics of a total dose of 20 mg CBD in sublingual drops was studied in six healthy subjects.^40^ In a representative experiment, the mean of the peak plasma concentration of CBD was 2 ng/mL at 130 min postdose. Similar values were obtained for a mixture of 20 mg THC+20 mg CBD applied either in sublingual drops or as aerosol; when applied through a nebulizer (10 mg THC+10 mg CBD), however, the peak plasma level was 9.5 ng/mL at 36 min after administration and the half-life of CBD in plasma was 66 min.

In a separate study with nine cannabis smokers, oromucosal application of low (5.4 mg THC+5.0 mg CBD) and high (16.2 mg THC+15.0 mg CBD) Sativex doses resulted in median peak plasma CBD concentrations of 1.2 ng/mL (range: 0.6–3.9 ng/mL) at 3.6 h (range: 1.0–5.5 h) postdose and 3.7 ng/mL (range: 2.0–20.5 ng/mL) at 4.5 h (range: 1.2–5.6 h) postdose, respectively^41^ (see also Stott et al.^42^).

The human skin permeation of CBD solutions was investigated *in vitro* and CBD concentrations as high as 6.1 mg per gram skin preparation could be achieved under certain experimental conditions.^43^ Various CBD formulations for transdermal and intranasal delivery have also been studied in rodent models.^44,45^

While first-pass metabolism could be avoided by rectal administration of suppository formulation of CBs, as it has been demonstrated for THC,^46,47^ relevant studies with CBD appear to be lacking.

Finally, an analysis of *in vivo* distribution of CBs in five postmortem cases indicated relatively high CBD concentrations in bile (up to 63 ng/mL) and muscle (up to 32 ng/g) tissues, and it was noted that the CBD content of the brain was unexpectedly high (up to 6.7 ng/g)^48^ (see also Fabritius et al.^49^). In these cases, however, factors influencing CB pharmacokinetics were unknown.

No information is available for tissue distribution of CBD or its metabolites in living humans and relevant animal studies are scarce. In rats, analysis of blood and brain 21.5 h after intragastric administration of 23.4 mg/kg of [^3^H]CBD in olive oil solution showed respective tissue concentrations of unchanged [^3^H]CBD of 20.2 ng/mL and 6.4 ng/g; the hepatic concentration of the drug was higher throughout the experiment and 20.8 ng/g was recorded even 84 h after treatment.^50^ In another test series also with rats, analysis of brain parts 5 min after administration of [^3^H]CBD (1 mg/kg i.v.) revealed an even distribution of the radiolabel (CBD+its unspecified metabolites) at about 1 ng/mg as initial peak concentrations throughout all brain regions examined.^51^

A recent study compared plasma and brain levels of CBD after oral or intraperitoneal (i.p.) administrations of the drug in Cremophor at 120 mg/kg to rats and mice.^52^ Following i.p. administration, mice had a higher CBD plasma level than rats (14.3 and 2.6 μg/mL, respectively), whereas oral dosing resulted in a similar peak plasma concentration in both species (∼2 μg/mL). Oral administration offered six times higher brain peak CBD concentrations in rats than in mice (8.6 vs. 1.3 μg/g). It was also noted that oral administration of CBD (120 mg/kg) dissolved in the micelle-forming Solutol resulted in enhanced absorption of the drug compared to the solution based on the emulsion-forming surfactant Cremophor as evidenced by higher peak concentrations and prolonged exposures in blood (3.2 μg/mL at 6 h and 2 μg/mL at 2 h, respectively) and brain (12.6 μg/mL at 4 h and 8.6 μg/mL at 4 h, respectively). The effects of cosolvents and excipients on pharmacokinetics, involving cytochrome P450 (CYP450) oxidases and P-glycoprotein efflux transporters, of lipophilic substances in general have been extensively investigated.^53,54^

It must be noted that none of the above studies reported on the metabolic fate of CBD and no information is available on the human pharmacokinetics of the metabolites. (For an early mouse study indicating slow elimination of unidentified polar metabolites, see Karler et al.^55^).

## Human Metabolites of CBD

The first demonstration of CB biotransformation in humans appears to be the study of Christiansen and Rafaelsen^56^ who in 1969 reported the separation by thin layer chromatography several polar CB derivatives from the urine of 10 volunteers drinking cannabis “tea”; however, no attempts were made to identify the substances. Subsequently, the use of sophisticated analytical techniques, especially GC-MS and, occasionally, reliance on synthetic standards for structure confirmation allowed the unequivocal identification of CB metabolites in humans (see below).

The first CBD metabolites to be identified were isolated from rat liver homogenate and their structures were determined as a primary alcohol derived from the oxidation at the allylic C-7 methyl group on the cyclohexene moiety (7-OH-CBD) and a secondary alcohol resulting from the oxidation of the central (C-3′′) methylene group of the pentyl side chain (3′′-OH-CBD; for numbering, see Fig. 1) in 1973.^57,57a^ Since then, biotransformation studies in mammals, including humans, using various types of CBD administration have indicated considerable species variability.^26,58,59^ All studies *in vivo* appear to have been restricted to the characterization of urinary CBD metabolites.

Being a good substrate of CYP450 mixed function oxidases, CBD undergoes extensive hydroxylation at multiple sites and further oxidations result in a complex metabolic pattern; altogether, some 100 CBD metabolites have been identified from various organisms.^26^ In general, the major metabolites of CBD were derivatives of CBD-7-oic acid (7-COOH-CBD) further oxidized at the side chain (Fig. 2a, b).

Following initial excretion studies,^32,58^ about 40 oxygenated human Phase I metabolites have been characterized.^26,58–60^ The urinary excretion profile of CBD metabolism has been reported only for a single case, which involved a dystonic patient chronically treated with 600 mg daily oral doses of CBD.^58,59^ The characterization of the metabolites relied on GC-MS analysis using an array of derivatization techniques. The chemical structures of the metabolites present in the urine larger than 1% of the total CB content are shown in Figure 2a, while minor metabolites found in trace amounts in the urine of the patient are listed by their short name in Figure 2b. The *O*-glucuronide conjugate of CBD was one of the most abundant urinary excretion products (13.3%), while the concentration of intact CBD was 12.1% of the total excreted CB. The two nonoxidized CB identified were Δ^8^-THC (structure not shown) and Δ^9^-THC, both presumably formed by cyclization of CBD, while cannabinol originating from aromatization of the Δ^8/9^-THC species was also present in the urine. A further nonoxidized CB, probably a cyclized monophenol, was also detected at 1.2% but its structure was not elucidated. Figure 2c lists five side-chain monohydroxylated CBD metabolites, which were recently identified in a human liver preparation *in vitro*^60^; all of them had been known from previous animal studies.^26^

Interestingly, inspection of the chemical structures of the abundant 7-COOH-CBD derivatives, which account for about the half of the metabolites identified in the above study, reveals the branched alkyl chain of (2*E*)-2-propylpent-2-enoic acid (Δ^2(*E*)^-valproate) embedded in the cyclohexenecarboxylic acid moiety of such acidic CBD metabolites. Δ^2(*E*)^-Valproate is the major active metabolite of valproic acid, and unlike the parent saturated acid, its anticonvulsant properties are not compromised by hepatotoxicity and teratogenicity, and is well tolerated in humans.^61^ Whether the 7-COOH-CBD metabolite species with a Δ^2(*E*)^-valproate-like structure are involved in the antiepileptic activity of CBD remains to be established.

The enzymatic processes responsible for the formation of the metabolites involve CYP450 oxidases, glucuronyl transferases and sulfotransferases, of which the CYP450 enzyme family has only been thoroughly studied.

Experiments *in vitro* with seven recombinant human CYP450 isoforms indicated 6α-OH-, 6β-OH-, 7-OH-, and 4′′-OH-CBD as main monohydroxylated metabolites.^60^ Specifically, CYP1A1 is involved in the formation of 6α/β-OH-, 7-OH-, and 1′′-OH-CBD; CYP1A2 is responsible for the formation of 6α/β-OH-CBD, as well as of 1′′-, 2′′-, 3′′-, and 4′′-OH-CBD species; CYP2C19 is involved in the formation mostly of 6α-OH-, 7-OH-, and 4′′-OH-CBD; CYP2D6 appears to be the main isoform responsible for the formation of 6α/β-OH-CBD as well as of 7-OH-, 4′′-OH-, and 5′′-OH-CBD; CYP3A4 efficiently catalyzes the formation of 6α/β-OH-CBD, although the 7-OH-, 2′′-OH-, 4′′-OH-, and 5′′-OH-CBD metabolites are also produced by this isoform; CYP3A5 is involved in the formation mainly of 6α/β-OH-CBD although the 7-OH-, 2′′-OH-, 3′′-OH-, and 4′′-OH-CBD metabolites are also produced. The role of CYP2A9 appears to be minor, but 6α/β-OH-, 7-OH-, 4′′-OH-, and 5′′-OH-CBD could be formed by this isoform.

Glucuronidation of CBD at the phenolic oxygen is a major Phase II biotransformation in humans,^59,62^ but hydroxylated metabolites of CBD may also be substrates. Sulfation of CBD species may also occur but such conjugates remain unknown. Studies should thus use appropriate hydrolysis before Phase I metabolite quantification.^63^

Finally, it should be mentioned that interindividual differences in the expression and function of CYP450 enzymes may considerably affect the pharmacokinetics of CBD and its metabolites, and this could be relevant in the therapeutic action and any possible adverse effects of CBD-containing preparations. For CBD, essentially no relevant information is available and we are aware only of one publication that deals with the genetic polymorphisms in CYP2C9 and CYP3A5 as related to THC metabolism in humans.^64^

### CBD-derived and metabolite-like substances

**FIG. 3.**
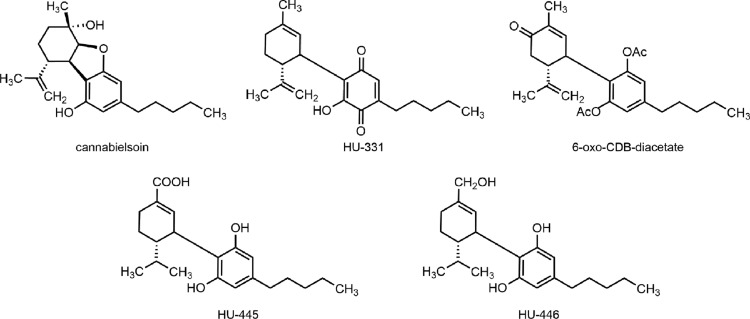
Chemical structures of CBD-derived substances of biological interest.

One of the interesting metabolites of CBD is cannabielsoin (Fig. 3), which was obtained first photochemically from CBD,^65^ and has not been isolated from humans but has been identified in guinea pigs.^66,67^ Furthermore, a hydroxyquinone derivative of CBD (HU-331; Fig. 3), which was again obtained first by synthesis,^68^ has been postulated to be a short-lived (re)active oxidative metabolite of CBD (see below).

Recently, the side-chain hydrogenated form of the minor human metabolite 7-COOH-CBD, that is, 8,9-dihydro-7-COOH-CBD (HU-445; Fig. 3), has been synthesized.^69^ The dihydrogenated product of the minor human metabolite 7-OH-CBD, that is, 8,9-dihydro-7-OH-CBD (HU-446; Fig. 3) has also been prepared and found to have anti-inflammatory properties *in vitro* with negligible affinity toward CB1 and CB2 receptors.^70^

## Biological Activity Studies with CBD Metabolites

### Studies *in vitro*

An early study^71^ on the inhibition of the binding of [^3^H]-5′-trimethylammonium-Δ^8^-THC to rat brain neuronal membrane homogenate by a series of CBs showed 7-OH-CBD (corresponding to 10-OH-CBD in the original publication) to be slightly less active than CBD (*K*_i_=94 and 73.1 nM, respectively; THC: *K*_i_=27 nm). Subsequent CB receptor binding studies^72–74^ with CBD enantiomers and their derivatives revealed that (−)-CBD (*natural* enantiomer) and its 7-OH and 7-COOH metabolites were devoid of receptor affinity (*K*_i_ > 10,000 nM); however, *synthetic* (+)-CBD was a modest receptor ligand with *K*_i_ values of 842 and 203 nM for the CB1 and CB2 receptor, respectively. The 7-OH and 7-COOH derivatives of (+)-CBD had high affinity to the CB1 receptor with respective *K*_i_ values of 5.3 and 13.2 nM; these two unnatural compounds also bound to the CB2 receptor with respective *K*_i_ values of 322 and 156 nM. Furthermore, while both CBD enantiomers proved to be equipotent full agonists of the type-1 vanilloid receptor (EC_50_=3500 nM), the 7-OH and 7-COOH metabolites of (−)-CBD were inactive.^72^ Furthermore, (−)-CBD and (+)-CBD, as well as the natural metabolite 7-OH-CBD, inhibited fatty acid amide hydrolase with IC_50_ values of 27.5, 63.5, and 34.0 μM, respectively, but the 7-COOH-CBD metabolite was inactive (IC_50_ > 100 μM); also, anandamide uptake by rat basophilic leukemia cells was inhibited by (−)-CBD and by its 7-OH metabolite with IC_50_ values of 22.0 and ∼50 μM, respectively, as well as by (+)-CBD (IC_50_=17.0 μM) but 7-COOH-CBD was inactive (IC_50_ > 50 μm).^72^

With regard to metabolic enzymes, not only CBD but also its 6α/β-OH, 6-oxo (not observed species in humans), and 10-OH metabolites effectively inactivated mouse liver microsomal CYP2C and CYP3A oxidases isolated from mice treated i.p. with 120 mg/kg CBD.^75^ The key role of the resorcinol moiety of CBD (and of its metabolites) in the inhibition has been established.^75,76^ A recent patent described 7-OH- and 7-COOH-CBD as anti-inflammatory substances in the mouse and as dose-dependent inhibitors *in vitro* of the generations of nitric oxide and reactive oxygen species as well as the production of TNF-α.^77^

The atypical quinone HU-331 was found to inhibit mouse hepatic microsomal CYP450 enzymes,^78^ CYP3A11 in particular,^79^ as well as induced apoptosis of splenocytes isolated from mice.^80^ Several lines of evidence have suggested that the inhibition (inactivation) is due to the adduct formation between this reactive quinone and the cysteine thiols at the active site of the enzyme. HU-331 also has antiangiogenic properties and is a selective inhibitor of topoisomerase II.^81,82^ Being more effective and safer than the prominent antitumor agent doxorubicin, HU-331 has been extensively investigated as a potential anticancer drug.^83^

### Studies in animals

There have been only a few *in vivo* investigations with selected monooxygenated metabolites. In the standard mouse “tetrade” test using i.p. injection of 20 mg/kg of the compounds, Fride et al. reported^84^ that CBD was inactive that reflects its lack of affinity to central CB1 and CB2 receptors (see Bisogno et al.^72^); 7-COOH-CBD caused slight hypothermia (−1.1°C) and a minimal inhibition of intestinal motility (defecation). Interestingly, unnatural (+)-CBD and its 7-OH and 7-COOH derivatives potently inhibited defecation indicating peripheral activity perhaps through a CB receptor-independent mechanism; furthermore, weak antinociceptive effects for 7-OH-(+)-CBD were also noted.^73,77^ As mentioned before, antinociceptive as well as anti-inflammatory effects in mice of 7-OH-CBD and 7-COOH-CBD have been described in a patent but these experiments used chemicals as noxious stimuli.^77^

Upon i.v. administration to rabbits, CBD (1 mg per animal) had no activity in lowering intraocular pressure but 10-OH-CBD (2 mg per animal) was effective with a slow onset of action; nevertheless, THC was more potent at the 1 mg dose in the study.^85^

Cannabielsoin (at ≤10 mg/kg i.v.) “showed no CNS activity” in rodents^,86^ and did not affect body temperature or pentobarbital-induced sleep time in mice.^66,67^ In rabbits, a 5 mg/kg i.v. dose of cannabielsoin reduced intraocular pressure; CBD had no effect at 10 mg/kg, while THC was active at 1 mg/kg.^87^

HU-331, which has been postulated to be a short-lived (re)active oxidative metabolite of CBD with CYP450 inhibitory properties,^79,80^ has been extensively investigated in rodents due to its anticancer activity.^83^

Finally, CBD as well as the synthetic 6-oxo-CBD-diacetate (Fig. 3), which could hydrolyze *in vivo* to the corresponding phenolic 6-oxo-CBD, showed anticonvulsant activity and prolonged pentobarbital sleep time when administered to mice at 200 mg/kg (i.p.)^88^; furthermore, CBD as well as the synthetic 10-OH-CBD-triacetate (structure not shown) reduced spontaneous motor activity in this trial. Although 6-oxo-CBD has not been detected in humans as a metabolite, its glycoside was identified in CBD-treated dogs.^89^

### Human studies

There are no publications describing the biological activity of CBD metabolites in humans.

### Interaction with other drugs

The pharmacological actions of CBD on receptors, ion channels, cellular uptake processes, and enzymes have recently been reviewed^9–11^ and are not reiterated here. Since CBD is often administered concomitantly with other medicines, for example, as an adjunct in the therapy of certain diseases, drug–drug interactions should be taken into account. What follows is a brief summary of such effects possibly having relevance in the clinical use of CBD. The contentious issue of CBD–THC interaction, however, is not discussed here (for a brief summary, see the relevant section in a recent review^90^).

The first pharmacological effect to be observed for CBD was, in fact, related to drug interaction. Already in 1942, it was noted by Adams^91^ that “cannabidiol, which is devoid of the marihuana effect upon man, showed the highest potency in this [mouse sleep prolongation] test” (that is extending the hypnotic effects of certain barbiturates); such a synergism was later shown to be related to the inhibition by CBD of mouse hepatic microsomal metabolism as demonstrated for phenazone (antipyrine).^92^ In young men with marijuana use experience, however, CBD at the low acute smoked dose of 0.5 mg/kg failed to affect the plasma level of secobarbital.^93^ Yet, a subsequent human pharmacokinetics study found that upon oral administration in a 6×100 mg daily dose regimen, CBD significantly increased the bioavailability and prolonged the elimination half-time of hexobarbital.^94^ It has also been proposed that the observed interference with drug metabolism *in vivo* could be due to one or more metabolites and not to the parent CB.^55^

It has repeatedly been demonstrated that CBD is not only a substrate but also an inhibitor of CYP450 enzymes, and thus, it could interfere with the metabolism of other xenobiotics, including THC and medicinal products.^27,34,76,90,95^ Moreover, prolonged CBD administration may induce specific CYP450 isoenzymes, as it has been shown in mouse liver CYP3A and CYP2B10^96^ as well as for human CYP1A1 *in vitro*.^97^ Also, 6α-OH-CBD, but not 6-oxo-CBD, was found to be an effective inducer of CYP2B10.^96^ The resorcinol moiety apparently plays a pivotal role in CYP450 induction.

The CYP450-mediated metabolism of anandamide *in vitro* by liver microsomes isolated from mice treated with CBD (120 mg/kg i.p.) was shown to be inhibited, but the physiological significance of this finding has not been explored.^98^ The involvement of CBD in the regulation of the endocannabinoid system has recently been reviewed.^10^

During a Phase I study with healthy male subjects, potential drug–drug interactions of THC/CBD oromucosal spray (Sativex, nabiximols) in combination of CYP450 inducers and inhibitors were assessed using various dose regimens.^99^ The antibiotic rifampicin, an inducer of CYP3A4 involved in the metabolism of CBD, significantly reduced the peak plasma concentration of CBD, while the antifungal ketoconazole, a CYP3A4 inhibitor, nearly doubled the peak plasma concentration of CBD; however, the moderate CYP2C19 inhibitor omeprazole, a proton-pump inhibitor used to treat gastroesophageal reflux disease, did not significantly alter the pharmacokinetics of CBD. The presence and role of CBD metabolites in the observed drug interactions have not been reported.

Recently, an 8-week trial studied the interaction of the anticonvulsant drug clobazam and CBD in 13 children with refractory epilepsy.^100^ Oral CBD treatment started with 5 mg/kg per day with a weekly increase of 5 mg/kg per day up to 25 mg/kg per day. It was observed that coadministration of the CB increased clobazam plasma level by 60%±80%, while the level of its active metabolite norclobazam increased by 500%±300%. This allowed the reduction of the initial average 1 mg/kg daily clobazam dose in most subjects thereby alleviating consequential side effects. Since both the bioactivation by demethylation of clobazam and the inactivation by hydroxylation of clobazam/norclobazam involve, respectively, CYP3A4 and CYP2C19 isoenzymes, which are also implicated in CBD metabolism, interactions with CBD and/or its metabolites in such combination therapies should be considered.

Furthermore, CBD has been shown to interact *in vitro* with P-glycoprotein efflux transporters involved in multidrug resistance, and thus, it may affect the pharmacokinetics of anticancer drugs.^101–103^ Also, the placental permeability in pregnant women who consume CBD-containing preparations may also be influenced.^104^ Again, no relevant information is available for the metabolites of CBD.

## Synthesis of CBD Metabolites

The identification of CBD metabolites has typically relied on mass spectral fragmentation patterns,^105^ and structural confirmation by synthesis was done only in a few cases; nevertheless, essentially all single-site modified CBD metabolites have been prepared. The aim of most of these syntheses was merely to verify the chemical structure of a metabolite and not to provide material for bioassays. The few exceptional studies were discussed in the preceding paragraphs.

Analytical characterizations of and synthetic methodologies for all five metabolites hydroxylated at the pentyl side chain were described in the early 1970s.^57,57a,106,107^ The syntheses of the epimeric 6α- and 6β-OH-CBD^108^ as well as the syntheses of the 7-OH-CBD^74,108–110^ and the nonhuman metabolite species 10-OH-CBD^109,111^ have been reported. The rabbit metabolite 8,9-diOH-CBD was obtained by incubating 8,9-epoxy-CBD^112^ with guinea pig hepatic microsomes.^113^ Ring- and side-chain hydroxylated derivatives, namely 3′′-OH-CBD,4′′,5′′-dinor, 4′′-OH-CBD, 4′′,6α-diOH-CBD, and 3′′,6-diOH-CBD,4′′,5′′-dinor, have also been produced in milligram quantities by microbial oxidation.^114^ The synthesis of the 7-COOH metabolite of CBD has been described.^74,115^ Synthesis of the side-chain terminal carboxylic acid (5′′-COOH, identified only in animals^116–119^) has also been described.^120^ A patent described the methyl ester of 2-COOH-CBD,3,′′4,′′5′′-trinor.^121^ The glucuronide of CBD has been prepared using a glucuronyltransferase.^122^ Cannabielsoin, a putative CBD metabolite may be obtained from CBD by several synthetic routes,^86,123,124^ as well as by biotransformation using tissue cultures of *C. sativa* and *Saccharum officinarum*.^125^ The 6-oxo-CBD derivatives, which may serve as precursors to the 6α/β-OH-CBD species, were prepared from CBD by allylic oxidation of the corresponding CBD derivative.^88,108^

Chemical syntheses of metabolites oxidized at multiple sites have not been published.

## Summary

Several drugs used in therapy are metabolically converted into active metabolites and interindividual variations in the generation and pharmacokinetics of such active species may cause variability in the response to treatment by different individuals.^126^ The use of relatively high daily doses of CBD in human clinical trials as well as in self-medicating patients is not uncommon. For example, in a 30-day CBD monotherapy study, an escalating oral dose reaching 1280 mg/day was administered.^127^ Although information is lacking, the metabolites formed from CBD are assumed to be present in the body at pharmacologically relevant concentrations. Pharmacological studies with such metabolites are scarce yet suggest interesting biological activities, which are unrelated or not directly related to CB receptors. Thus, intriguing questions arise:

Could any of the pharmacological effects observed for CBD be attributed to its metabolites?

Are there any drug–drug interactions that affect the outcome of the therapeutic effects of other, non-CB medicines used concomitantly with CBD?

Could any of the metabolites be used as templates for the development of novel therapeutic agents?

The pharmacological characterization of CBD metabolites both *in vitro* and *in vivo* is timely and necessary to shed light on the multifaceted, perplexing, or sometimes even contradictory biological properties observed for the parent CB. The understanding of the clinical significance of these abundant metabolites in the proven therapeutic effects of CBD-containing preparations warrants further studies.

## Supplementary Material

Supplemental data

## Abbreviations Used

CB: cannabinoid
CBD: cannabidiol
CBDA: cannabidiolic acid
i.p.: intraperitoneal
i.v.: intravenous
THC: tetrahydrocannabinol
TNF-α: tumor necrosis factor alpha
