# Supplementary material for: Human Metabolites of Cannabidiol: A Review on Their Formation, Biological Activity, and Relevance in Therapy
Source: Cannabis Cannabinoid Res. 2016 Mar 1;1(1):90–101. doi: 10.1089/can.2015.0012 (PMC5576600; doi:10.1089/can.2015.0012)
Supplement: Supplemental data [file Supp_Data.pdf]

Supplementary Data

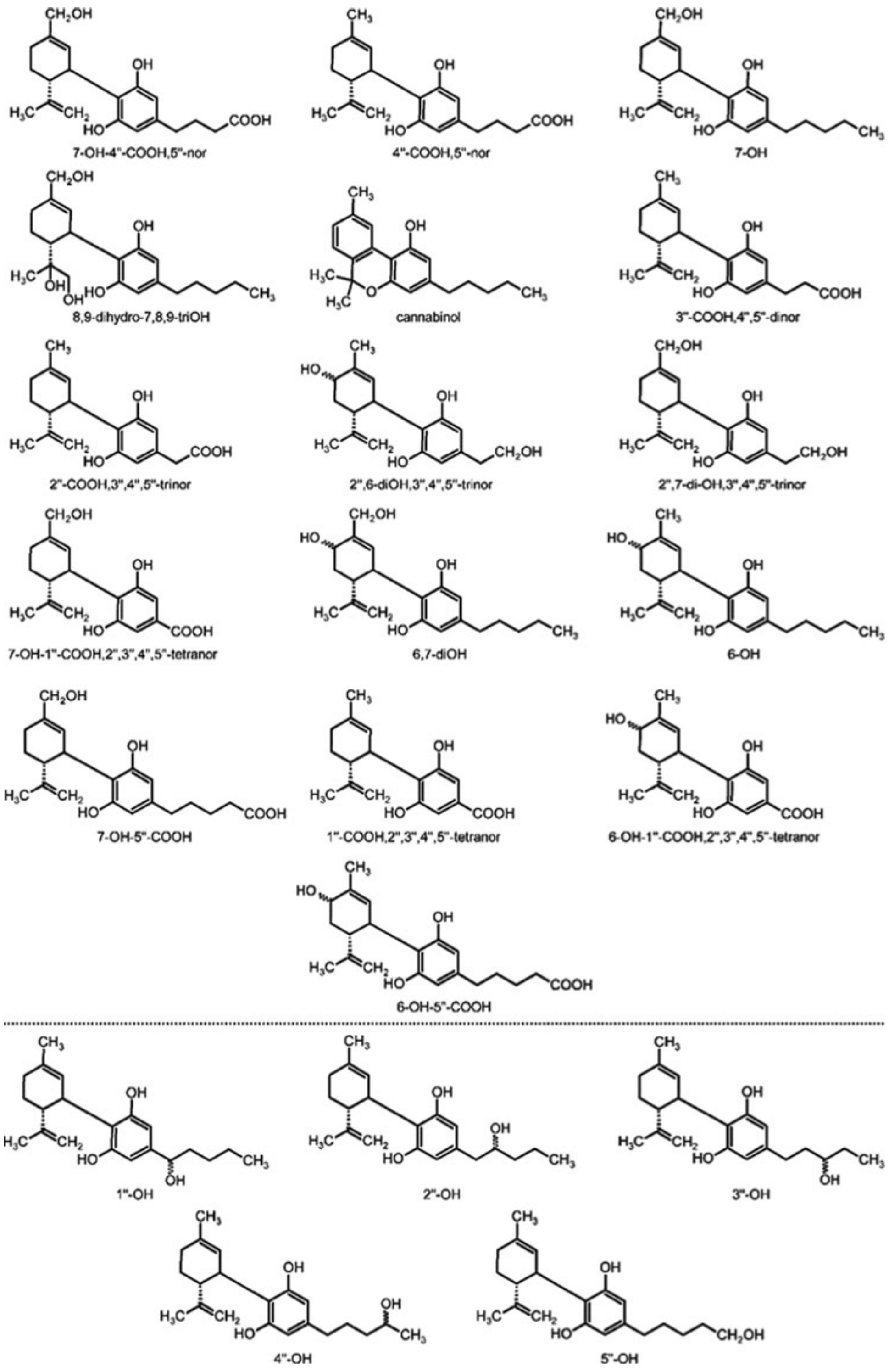

SUPPLEMENTARY FIG. S1. Above dotted line: chemical structures of minor (<1%) human CBD metabolites identified by Harvey and Mechoulam[1] in anhydrolyzed human urine of a dystonic patient chronically treated with CBD. The excreted products are listed according to their relative peak areas in the gas chromatogram (decreasing from upper left to lower right). Below dotted line: Chemical structure of human CBD metabolites identified in human liver microsomal preparation.[2] Wavy bonds indicate unknown stereochemistry at the stereogenic center.

Supplementary References

1. Harvey DJ, Mechoulam R. Metabolites of cannabidiol identified in human urine. Xenobiotica. 1990;20:303–320.
2. Jiang R, Yamaori S, Takeda S, et al. Identification of cytochrome P450 enzymes responsible for metabolism of cannabidiol by human liver microsomes. Life Sci. 2011;89:165–170.
